# Effects of repetitive Iodine thyroid blocking on the foetal brain and thyroid in rats: a systems biology approach

**DOI:** 10.1038/s41598-020-67564-8

**Published:** 2020-07-02

**Authors:** David P. A. Cohen, Mohamed Amine Benadjaoud, Phillipe Lestaevel, Dalila Lebsir, Marc Benderitter, Maâmar Souidi

**Affiliations:** 1 Institut de Radioprotection Et de Sûreté Nucléaire (IRSN), PSE-SANTE/SESANE, 92262 Fontenay-aux-Roses, France; 2 Institut de Radioprotection Et de Sûreté Nucléaire (IRSN), PSE-SANTE/SERAMED, 92262 Fontenay-aux-Roses, France

**Keywords:** Machine learning, Microarrays, Statistical methods, Developmental neurogenesis, Biochemical networks

## Abstract

A single administration of an iodine thyroid blocking agent is usually sufficient to protect thyroid from radioactive iodine and prevent thyroid cancer. Repeated administration of stable iodine (rKI) may be necessary during prolonged or repeated exposure to radioactive iodine. We previously showed that rKI for eight days offers protection without toxic effects in adult rats. However, the effect of rKI administration in the developing foetus is unknown, especially on brain development, although a correlation between impaired maternal thyroid status and a decrease in intelligence quotient of the progeny has been observed. This study revealed distinct gene expression profiles between the progeny of rats receiving either rKI or saline during pregnancy. To understand the implication of these differentially expressed (DE) genes, a systems biology approach was used to construct networks for each organ using three different techniques: Bayesian statistics, sPLS-DA and manual construction of a Process Descriptive (PD) network. The PD network showed DE genes from both organs participating in the same cellular processes that affect mitophagy and neuronal outgrowth. This work may help to evaluate the doctrine for using rKI in case of repetitive or prolonged exposure to radioactive particles upon nuclear accidents.

## Introduction

During a nuclear emergency, large amounts of radioactive particles may be released and may contaminate the environment and populations. Isotopes present in the atmospheric discharges include radioactive iodine-131. To prevent health consequences, one of the main protective methods is the Iodine Thyroid Blocking (ITB) agent which aims to saturate the thyroid gland with non-radioactive iodine and thus avoiding the fixation of radioactive iodine (Wolff-Chaikoff effect)^1^.

However, the recent 2011 Fukushima Daiichi disaster suggested that the World Health Organization’s “ITB guidelines”^2^ that so far supported a single intake of 130 mg potassium iodide (KI) tablet for individuals older than 12 years, cannot adequately protect populations in case of prolonged (beyond 24 h) or repeated exposure. In such situations, a second intake of stable iodine is possible, although the “ITB guidelines” so far give no indication on how to implement such repeated administration of KI^2,3^. Therefore, more knowledge about repetitive stable iodine administration is essential to prevent the side effects of repeated ITB on specific sensitive sub-populations such as pregnant women, foetusses, and elderly^3^.

Recently, preclinical data, obtained by our group, have showed that repeated ITB procedure with a daily administration of 1 mg/kg/day^4,5^ for eight days of KI does not induce adverse outcome (AO) in adult rats^6,7^. However, for higher risk groups such as pregnant women and unborn children, a negative impact may be expected, such as hypothyroidism and related CNS impairment, decrease in TSH and T_4_-thyroid hormone, and change in behaviour^8–12^. Repeated ITB administration for eight consecutive days in gestational rats, as a model for pregnant women, led to AO effects, such as changes in expression of thyroid hormone-responsive genes in the brain and motor coordination, in the offspring^8,13^. Here, we hypothesized that transient hypothyroidism caused by the dose regimen for repeated ITB in pregnant rats^8^, results in congenital hypothyroidism in the foetus and consequently to neurotoxicity.

**Figure 1:**
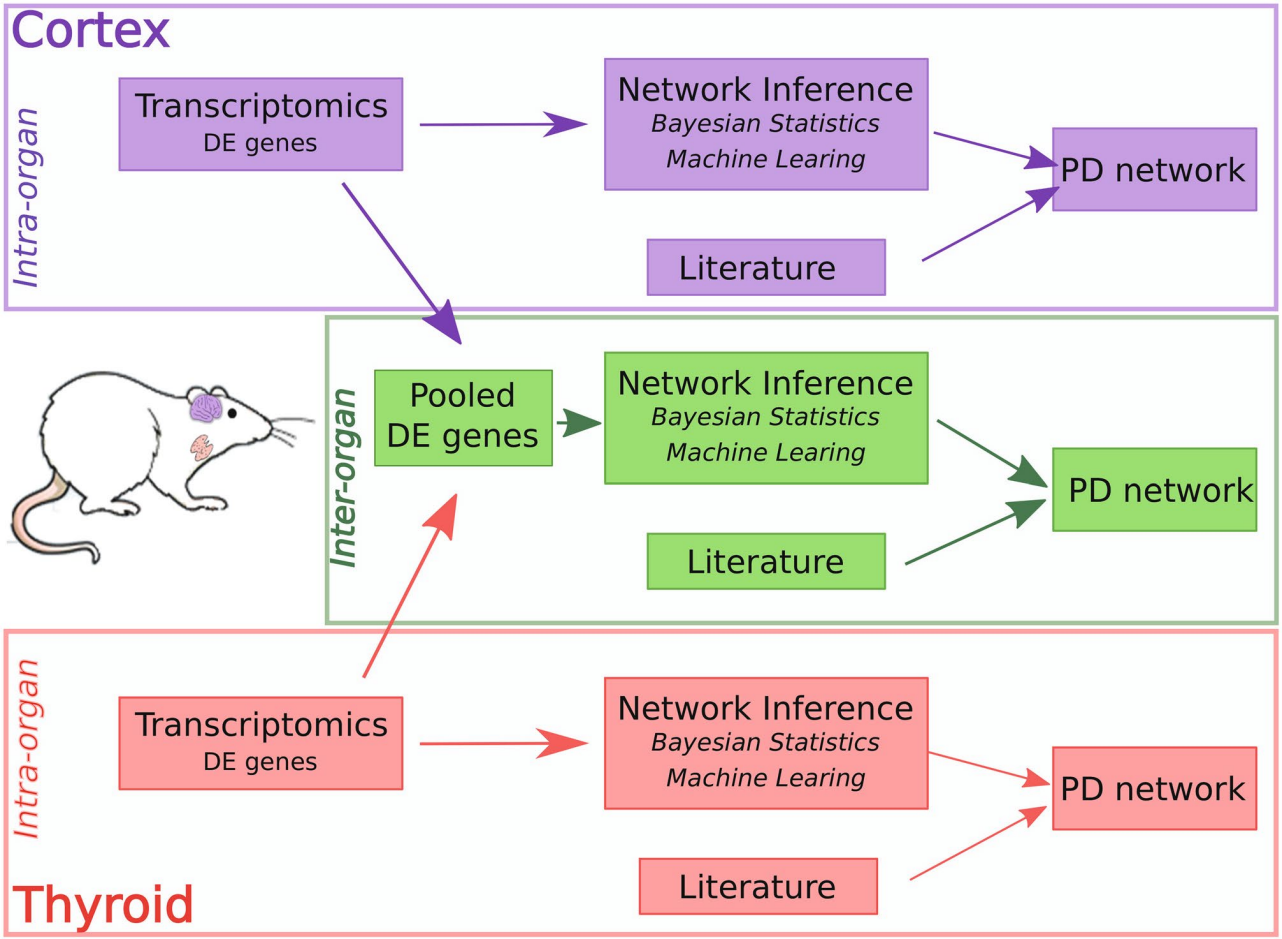
A diagram depicting the workflow that has been followed in this study. Two groups consisting of pregnant rats received either KI or saline solution for eight days starting at nineth gestational day until the 17th gestational day. Transcriptomics was performed on tissue from the cortex (brain) and the thyroid from the progeny, 51 days after birth. After analysis of differentially expressed (DE) genes networks are statistically inferred using either the ShrinkNet R-package or Machine Learning method using the MixOmics R-package. Based on both networks, a process descriptive (PD) network is manually constructed using literature and the above-mentioned networks, to elucidate putative molecular mechanisms that are associated with the AO of the CNS of the progeny during repetitive administration of KI by pregnant mother rats. This procedure has been performed for each organ (thyroid and cortex; boxes labelled intra-organ) and for inter-organ for which DE genes from both organs have been pooled together and subsequently the same procedure has been performed on the pooled genes.

In this study, a gestational Wistar rat model has been subjected to repetitive KI administration for eight days during the period of brain and thyroid development of the foetus. A systems biology approach was used to identify a putative molecular mechanism that could explain the effect of the iodine thyroid blocking agent KI on the thyroid and brain cortex. Two groups consisting of pregnant rats received either KI or saline solution for eight days starting at nineth gestational day until the 17^th^ gestational day. To associate genes with the AO impacting the Central Nervous system (CNS) previously observed by Lebsir and colleagues^8^, transcriptomics was applied to identify differentially expressed (DE) genes in the thyroid and the cortex in the progeny of the rats belonging to either the KI-treated or the control (saline-treated) group, 51 days post-natal (Fig. 1). In contrast to the study of Lebsir and colleagues in which targeted genes were considered^8^, this study proposes a large-scale gene screening in order to explore, in a more exhaustive manner, putative mechanistic interactions between genes of the cortex and thyroid. From these DE genes, correlations between them were inferred using two different statistical methods. In addition, to determine a putative mode of action, a Process Descriptive (PD) diagram has been constructed using the identified DE genes and their correlation data enriched with data obtained from the PubMed database. The PD diagram represents molecular mechanisms^14^ and identifies genes belonging to key events that may be associated with the AO. In addition, identification of causal relationships between genes from the thyroid and the cortex was explored.

## Results

### Gene expression profile for the thyroid and cortex

**Figure 2:**
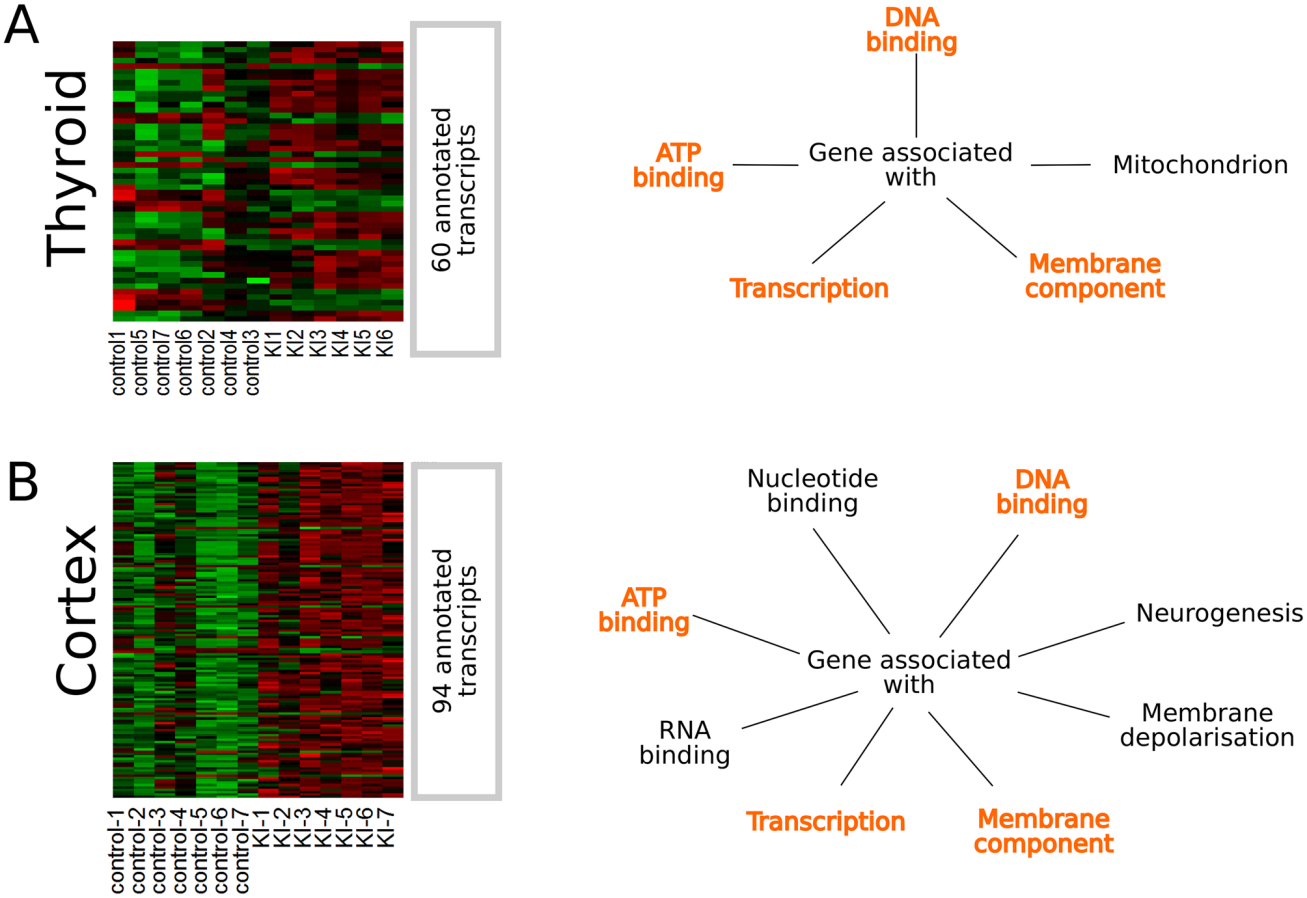
Heat map of significant differentially expressed (DE) genes and their associated function or cellular localisation isolated from progeny rats after 30 days of weaning in the thyroid (**A**) and the cortex (**B**). The progeny has been divided into two groups; one group exposed *in* utero to KI treatment while the control group was not exposed to KI i*n utero.* (**A**) shows a heat map of DE genes and some of their associated function and cellular localisation of genes obtained from the thyroid. (**B**) shows a heat map of DE genes and some of their associated function and cellular localisation of genes obtained from the cortex. The orange font indicates common associated functions both present in the thyroid and the cortex.

Gene expression analysis was performed on brain cortex and thyroid of the progeny from the two groups of pregnant rats, (KI-treated group and control group (saline-treated), respectively, 51 days post-natal. Compared to the saline-treated group, 60 and 94 genes were found to be differently expressed for the thyroid and cortex from the KI-treated group, respectively (t-test, Q-value < 0.05) (Fig. 2). A number of biological functions associated with these genes have been retrieved by querying the DAVID database^15^, including site-specific DNA binding, ATP binding, and transcription for the thyroid (Fig. 2A right-hand panel and Supplementary data Table 1), and transcription regulation, DNA, RNA and ATP binding, nucleotide binding and neurogenesis for the cortex (Fig. 2B right-hand panel and Supplementary data Table 2). These lists of transcripts were used to construct a network to attempt finding a molecular mechanism that describes the effect of KI on thyroid and/or cortex.

### Network inference for the thyroid upon repetitive KI treatment

**Figure 3:**
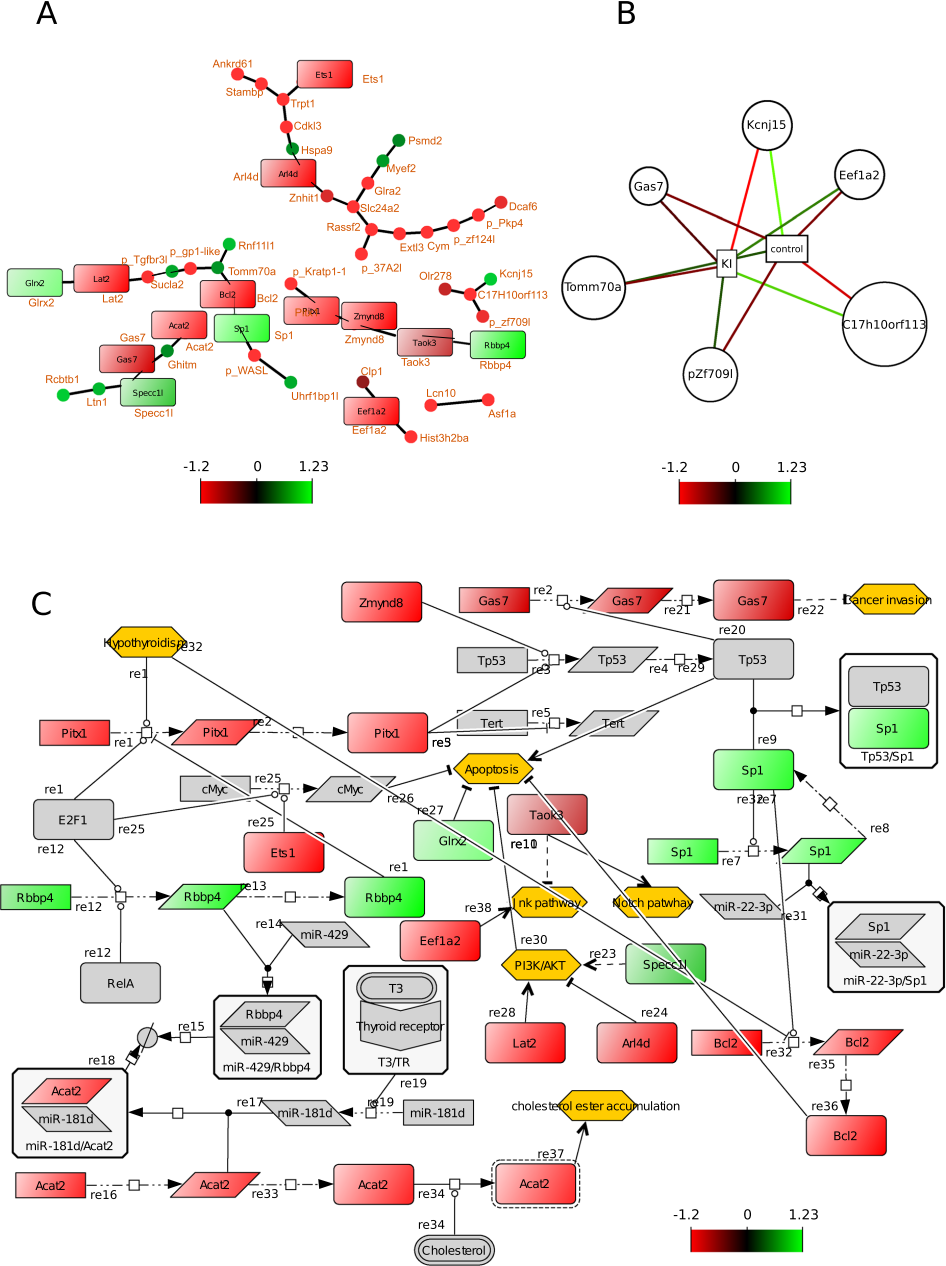
Selected diagrams show correlations between genes from the thyroid. The colour of the nodes indicates up- or down-regulation. Ochre coloured hexagons show phenotypes/biological processes that are modulated by the network. (**A**) shows a undirected network of DE genes using Bayesian simultaneous-equation model with global–local shrinkage ^53^. The nodes (closed circles and squares) represent genes and edges represent correlation between genes. The square nodes are genes that can be found in the process descriptive network as well (**C**). (**B**) shows the results of sPLS-DA: genes that contribute most to either the control group or the KI exposed group, respectively. These genes will be used as a starting point for constructing the PD network (**C**). (**C**) shows a process descriptive network that represents molecular mechanism^14^. Squares that are colour coded shows the fold changes of DE genes. The grey shapes are molecules obtained from published articles. All edges have been previously described in the literature.

Bayesian statistics showed that the 60 genes from the thyroid could be connected into one network that consists of four major sub-networks. In these sub-networks, there are few genes (nodes) that are connected to multiple other nodes in contrast to many nodes that connect only to a few (two other) nodes (Fig. 3A). This inferred network contained indications for constructing a PD network, such as which proteins or genes might interact with other molecules, and how many different (un-related) processes might be present.

Genes used for constructing the PD network were prioritised by applying the sPLS-DA method to obtain genes that contributed the most to the difference in phenotypes (control and KI exposed groups). Notably, all genes selected by sPLS-DA except for *tomm70a* and *kcnj15* were under-expressed in the KI-exposed group (Fig. 3B). The combination of the results of Bayesian statistics and sPLS-DA was used as input for constructing the PD network. For example, the gene *gas7* was identified using sPLS-DA as a contributor that distinguishes the control and treated groups. This gene was used as a starting point for creating the PD network. Database search in PubMed linked this gene or its corresponding protein to p53 and cancer invasion. The former was linked to SP1 whose gene was found in the inferred Bayesian network. Systematically all genes that have been identified by sPLS-DA (*gas7, kcnj15, eef1a2, c17h10orf113, pzf709l,* and *tomm70a*) were used as key words for PubMed database search.

The PD network contained 45 chemical species including genes, proteins, RNAs, anti-sense RNA molecules and 37 edges that have been annotated with references to the original scientific articles (Fig. 3C). The annotations can be found back in the “reaction notes” using the CellDesigner software. The network was based on 24 unique articles retrieved from the PubMed database. The PD network displayed genes and their corresponding proteins (coloured-code boxes) associated with functions including ATP and DNA-binding, transcription, as well as cellular locations such as cell membrane and mitochondrion (Fig. 2A). The grey-coloured molecules including genes and proteins were retrieved from the literature and formed together with the DE genes and their products a biochemical reaction network that represented molecular mechanisms in the thyroid. Gene expression data was applied (colour coded genes and their products) to this network which allowed better understanding of the effect of KI on gene expression in the network. Certain biological processes (ochre coloured hexagons), including the AKT, Notch and Jnk pathways, may be less active as the expression of *lat2*, *arl4d*, *taok2* and *eef1a2*, respectively, was reduced (Fig. 3C). In contrast, the genes *sp1* and *glrx2* were up-regulated which may result in decreased apoptosis as Glrx2 has a direct negative effect on apoptosis, while Sp1 traps P53 into a complex that prevents P53 from activating the apoptosis pathway (Fig. 3C).

### Network inference for the cortex upon repetitive KI treatment

For the cortex, we applied the same strategy. Transcriptomics showed 94 DE genes between KI-treated and control groups, and these genes were associated with biological functions including ATP-, DNA- and RNA-binding, transcription, neurogenesis, and association with the membrane (Fig. 2B).

**Figure 4:**
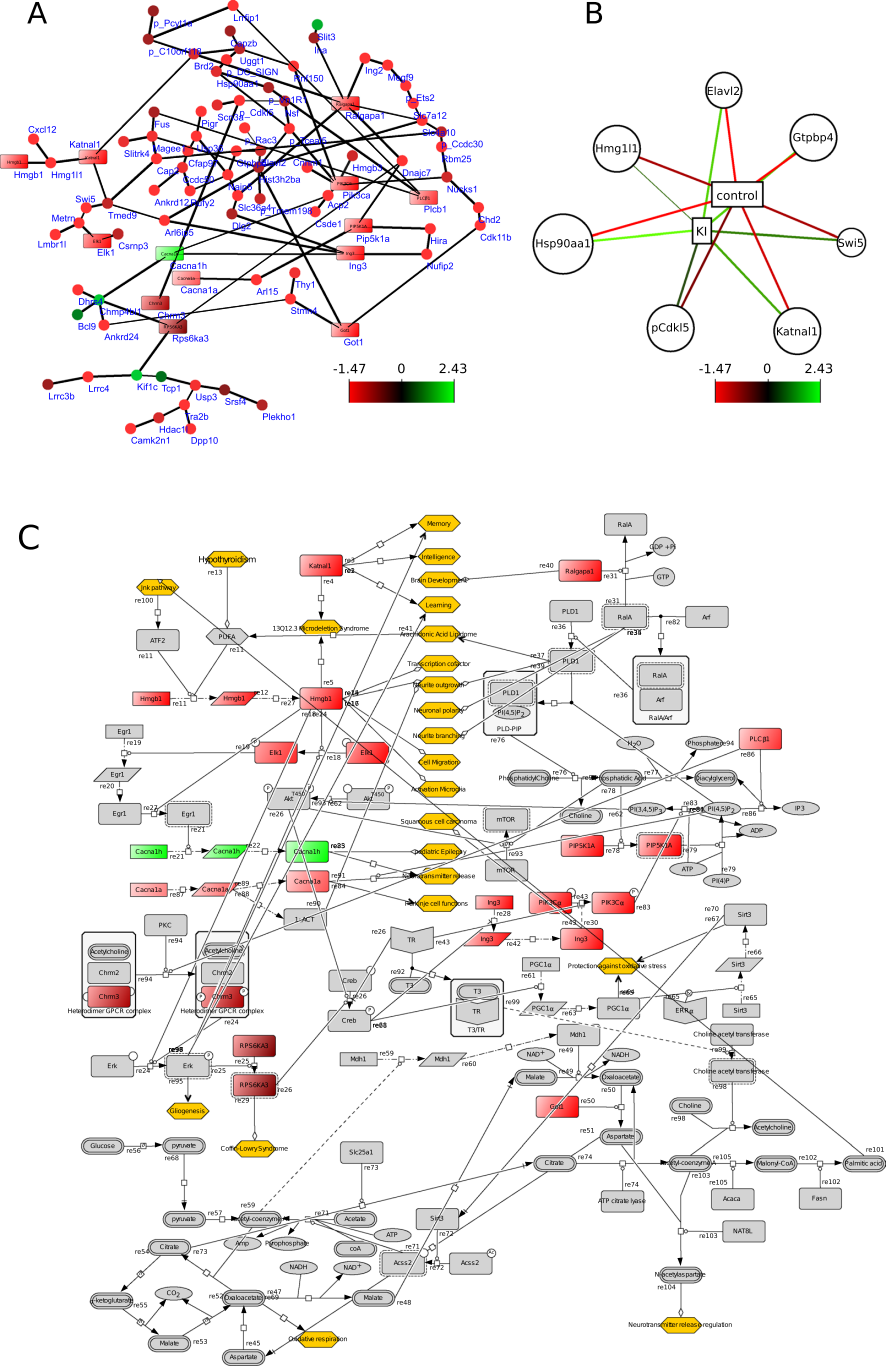
Selected diagrams showing correlations between genes from the cortex. The colour of the nodes indicates up or down regulation. Ochre coloured hexagons show phenotypes/biological processes modulated by the network. (**A**) shows a undirected network of DE genes using Bayesian simultaneous-equation model with global–local shrinkage^53^. The nodes (closed circles and squares) represent genes and edges represent correlation between genes. The square nodes are genes that can be found in the process descriptive network as well (**C**). (**B**) shows the results of sPLS-DA: genes that contribute most to either the control group or the KI exposed group, respectively. These genes will be used as a starting point for constructing the PD network (**C**). (**C**) shows a process descriptive network that represents molecular mechanism^14^. Squares that are colour coded shows the fold changes of DE genes. The grey boxes are molecules obtained from published articles. All edges have been previously described in the literature.

The inferred network (Fig. 4A) showed one highly connected network that is scale-free: few nodes that have many connections (hubs) and there are many nodes with a few connections indicating this network is not a random network^16^. The hubs in this network corresponded to the genes *hist3h2ba, ralgapa1, pik3ca and chmp4bl1*, which are involved in DNA binding, GTPase activation, ATP binding, and vacuolar transport, respectively (Supplementary Table 3). The genes *elavl2, gtpbp4, hmg1l1* (strong homology with hmgb1)*, hsp90aa1*, *katnal1, swi5,* and the predicted gene *cdkl5* were selected by sPLS-DA as strong contributors to differentiate between the control and the treated group and were all under-expressed in the KI group (Fig. 4B). They were associated with nucleotide binding, GTPase activity, DNA-binding, neurogenesis, ATP-binding, DNA repair (Supplementary Table 3).

The PD network was constructed from the network inferred from Bayesian statistics and the genes identified by sPLS-DA. The PD network from the cortex contained 130 chemical species including genes, proteins, RNA, anti-sense RNA molecules and 92 edges/biochemical reactions that were annotated within the “reaction notes” (Fig. 4C). The network was based on 44 unique articles retrieved from PubMed. The PD network displayed genes and their corresponding protein (coloured-code boxes) associated with biological functions including ATP- and DNA-binding, transcription, neurogenesis, as well as cellular locations such as cell membrane, mitochondrion (Fig. 4A). The grey-coloured genes and proteins were retrieved from the literature and together with the DE genes and their gene products they formed a biochemical reaction network that represented molecular mechanisms in the cortex. Gene expression data was applied to this network (colour coded genes and their products) which allowed better understanding the effect of KI on gene expression in the network. Certain DE genes including *katnal1*, *hmgb1*, *cacna1a* and *ralgapa1* modulate the biological processes (Ochre coloured hexagons) associated with memory, intelligence, learning, neurite outgrowth and branching, transmitter release and brain development (Fig. 4C). Both hmg1l1 and katnal1 were identified as strong contributors to discriminate control and treated groups.

### Between-organ integrative network: thyroid and cortex upon repetitive KI administration

**Figure 5:**
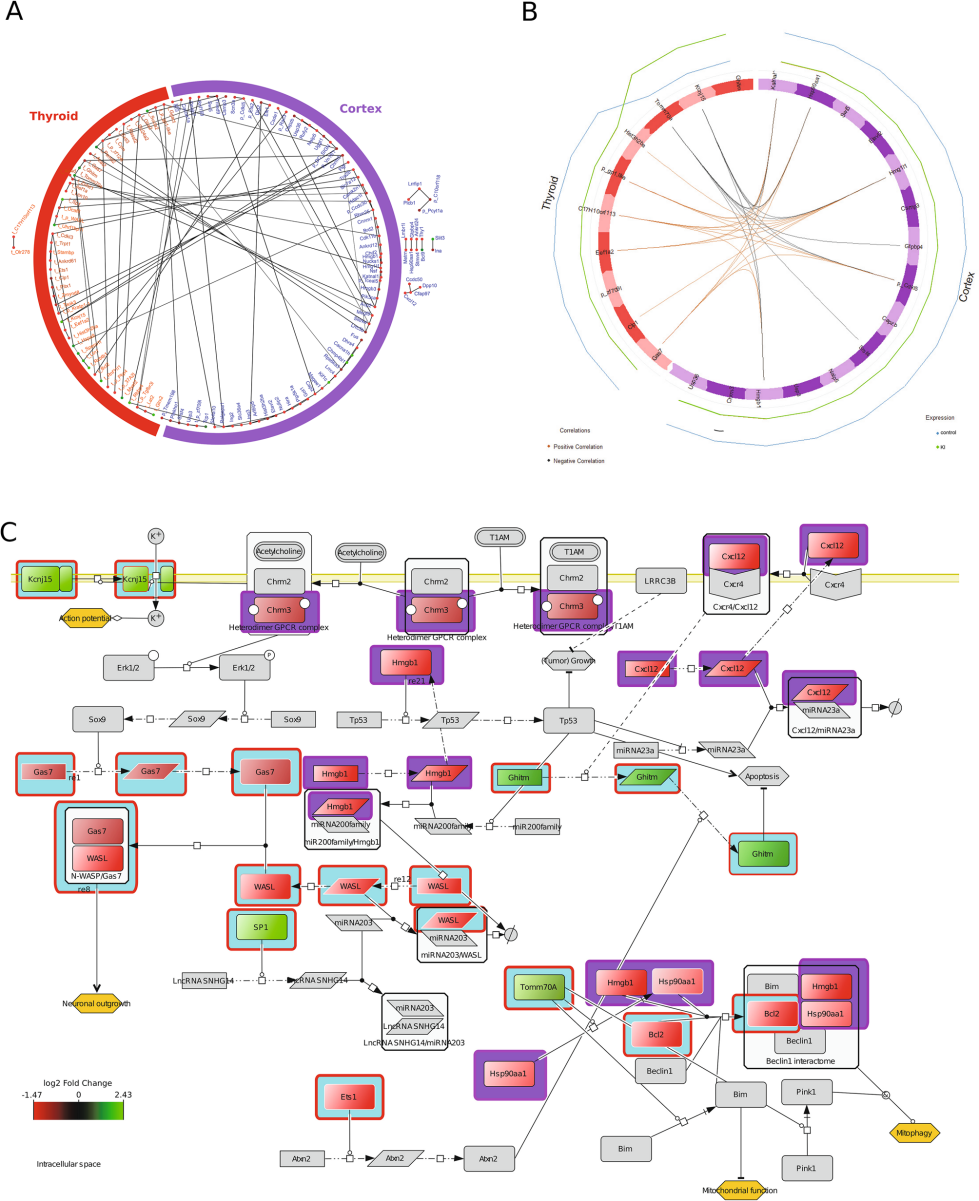
Different networks showing interaction between DE genes from both thyroid and cortex. (**A**) shows a ShrinkNet network of DE genes obtained from thyroid and cortex respectively. There are edges that show correlation between expression of genes within the same organ (thyroid or cortex), as well as edges showing correlation between genes from both organs. In addition, there are genes present from one organ that either do not have edges to genes of the other organ or do not have edges to genes that have edges to genes of the other organ (nodes that are outside the circle). (**B**); The DIABLO inferred gene network is represented as a circle where the red part of the circle represents the thyroid with genes that contribute strongly to the separation of the control group and the KI exposed group. The purple part of the circle represents the cortex with genes that contribute strongly to the separation of the KI group and the control group. The edges show correlation (with a cut-off of r = 0.82) between genes that can be either negative (black colour) or positive (orange colour). These genes can be used as a starting point for creating the PD network (**C**). (**C**); A process descriptive network shows DE genes that are obtained from the thyroid (light blue background) and cortex (purple background), respectively. The information about the interaction between these genes and their corresponding proteins and the interaction with other molecules is obtained from published articles. This figure demonstrates molecular mechanisms between genes from two different organs that impact neuronal outgrowth, and action potential in neurons and mitochondrial function and mitophagy (Ochre coloured hexagons) within the cell.

It was previously shown that thyroid hormones are crucial for brain development^17^. Here, we assessed whether gene expression profiles of the two organs (thyroid and cortex) were correlated, by applying Bayesian statistics using ShrinkNet, and DIABLO framework using mixOmics. The ShrinkNet inferred network showed both “between” and “within” organs correlations (Fig. 5A) and the inferred network obtained by DIABLO connected the selected genes in both organs which maximize the “between-organ” transcriptomic expression correlation (Fig. 5B).

To “validate” the results of the ShrinkNet and DIABLO frameworks, a PD network was constructed (Fig. 5C). One process that was of particular interest was the activation of the Chrm2/3 complex by acetylcholine that led to activation of the ERK transcription factor (TF) which in turns increased transcription of *Sox9*. The Sox9 TF induced the transcription of *gas7* and the GAS7 protein, associated with the WASL protein (complex), affected the neuronal outgrowth. The gene *chrm3* and the genes *gas7* and *wasl* were differentially expressed in cortex and thyroid, respectively. In addition, the genes *tomm70a, bcl2¸*and *hsp90aa1, hmgb1* from thyroid and cortex, respectively, were implicated in mitochondrial function and mitophagy.

## Discussion

The Fukushima disaster with repetitive atmospheric releases of radionuclides has revealed the limitation of a strategy based on a single ITB administration, which may not be sufficient to protect the thyroid. Repetitive or multiple doses of ITB have been shown to protect against prolonged exposure of ^131^I in adults^18^. However, for groups at higher risk *i.e.* pregnant women and unborn children, a negative impact may be expected such as hypothyroidism and related CNS impairment, decrease in TSH and T4, as well as a change in behaviour^8–12^. The molecular mechanisms that drive these AO are currently unknown and need further investigation to propose adapted/optimised KI prophylaxis. The application of systems biology may provide additional insights on the effect of KI and may be able to identify associated molecular mechanisms^19–21^.

In this study, we focussed on the thyroid and cortex as these organs are the major target organs of (ITB-induced) hypothyroidism in the mother during embryonal development of the progeny^22,23^. Indeed, for both cortex and thyroid, distinct gene expression profiles between the control and KI-treated groups were observed even at 51 days after birth, while in adult rat no difference in gene expression between the control and KI-treated groups have been observed^6^. We hypothesized that the observed late gene expression profile (Fig. 2) is a result of KI-disturbed foetal development. Actually, it was previously shown that ITB treatment in the mother during pregnancy could affect the progeny^24–27^.

The gene expression profile for the cortex has shown that DE genes are associated with DNA binding (*hmgb1, hmgb3, hmg1l1, ing2, csde1, lrrfip1, hist3h2ba*), transcription (*brd2, ing3, elk1, hdac1l, lrrfip1*), and neurogenesis (*ina, metrn, slit3*)*.* In the thyroid, DE genes are associated with transcription (*sp1, ets1, pitx1*), DNA binding (*sp1, ets1, hist3h2ba, pitx1*), as membrane components (*stambp, olr278, kcnj15, extl3, lat2, ghitm, slc24a2, bcl2, glra2, gabbr1*)*.* However, some DE genes, identified in the above functional groups, are critical for brain development^28–31^. In order to understand how the above listed genes are interconnected, and to identify the cellular processes that may be deregulated during ITB treatment, a network construction based on DE genes was performed. Indeed, network construction is a well-established method to asses patho-physiology^32^ and can provide new insights in the etiology of diseases^21^.

We took advantage of different methods for inferring a network, *i.e.* Bayesian statistics (ShrinkNet) and sPLS-DA/Diablo. Although these networks are undirected (no causal relationship), the topology of the networks may provide information on how robust the cellular processes may be. For the thyroid, the four sub-networks may represent four different biological processes that may contribute to the same dysfunction. In contrast, the network of the cortex showed one main network with few hubs (nodes with many connections). Hubs are essential nodes and targeting these nodes impacts the network significantly while non-hub nodes are less important and mutations of those nodes have less impact on the network^16^. Due to its complexity (many more connections compared to the thyroid network), it is more difficult to find genes that may lead to synthetic lethality^33^ and therefore the cortex network is more robust against deregulation of homeostasis than the thyroid one. Because the thyroid network is less robust, it is easier to modulate this network, *i.e.* a low-dosage KI may have a major impact on thyroid hormone synthesis, which may have in turn a major effect on the development of the cortex during embryonal development^34^.

The obtained networks showed connections between genes in terms of statistical correlations and thus did not show causal effect on the surrounding genes, the PD networks allowed a plausible and biochemical interpretation, hence providing a more mechanistic view compared to the inferred networks. The PD network of the thyroid suggested that the DE genes may affect apoptosis, although it is not clear if this is either positive or negative, since Tp53 (apoptosis activator) is inhibited by the DE genes *pitx1, rbpp4 and sp1*. On the other hand, KI treatment inhibited the PI3K/AKT pathway and *bcl2,* which are known as apoptosis inhibitors^35,36^. The PD network suggests that apoptosis is a target of repetitive ITB; KI-induced hypothyroidism can activate apoptosis through *bcl2*^37^*.* This result was unexpected after the initial gene profiling approach since no apoptotic function arose among the listed cellular processes. It has been demonstrated before that the excess of KI induced the activation of the molecular mechanisms associated with apoptosis^38,39^. Furthermore, AKT was found to be critical in the development of the brain upon iodine deficiency-induced hypothyroidism^40^.

In the cortex PD network, the genes *hmgb1* and *katnal1* have many direct arches with phenotypes that are associated with memory, learning, brain development, etc. These direct connections have been described before, however, the exact mechanisms behind these observations are still unknown. In addition, the up-regulated gene *cacna1h* is involved in childhood absence epilepsy^41^, the down-regulated gene *cacna1a* is involved in the activation of muscarinic M1-class receptors and neurite outgrowth^42,43^, and the down-regulated *got1* gene may negatively impact the synthesis of acetylcholine and therefore affect neuronal communication^44^. The PD diagram shows putative mechanisms on how these DE genes, especially *katnal1* and *hmgb1*, may impact brain development and its function upon ITB treatment of pregnant rats. Indeed, Lebsir and colleagues have shown that multiple administration of KI during pregnancy led to alterations of behaviour in the progeny of rats^8,13^.

**Figure 6:**
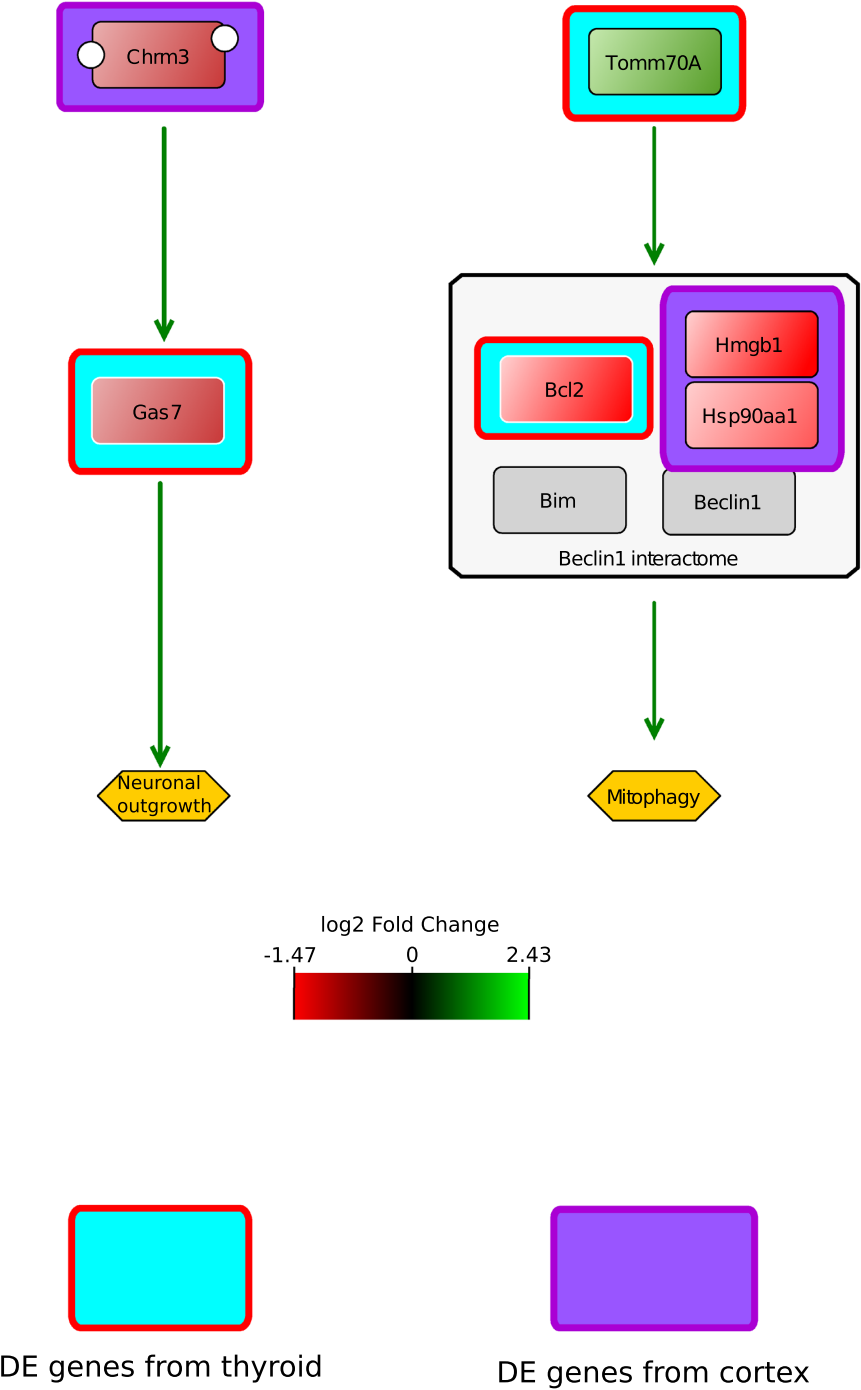
A simplified diagram derived from Fig. 5 showing the positive effect of DE genes that have been identified using the sPLS-DA/DIABLO platform on neuronal outgrowth or mitophagy. Their effect is based on based on their involvement in the biochemical reaction network (PD diagram, Fig. 5). The gene expression is colour coded and added to the genes. In case of the genes *chrm3* and *gas7*, both are under-expressed and this may result in diminished neuronal outgrowth as during homeostatic conditions both genes have an activating effect on neuronal outgrowth. The gene *tomm70a* is over-expressed and this gene has a positive effect on the formations of the Beclin1 interactome which may have an activating effect on mitophagy during homeostatic conditions. However, three DE genes are under-expressed and this may result in no interactome formation. Alternatively, *tomm70a* is over-expressed to compensate for the loss of the Beclin1 interactome.

Thyroid and cortex developments are connected during foetal development. Repetitive ITB administration occurring during this period leads to a joined impact on these two organs. This motivated us to connect the DE genes from both the cortex and the thyroid into one network, which suggests (i) cortex to thyroid connection through *chrm3* and *gas7* that induces neuronal outgrowth^45–47^; (ii) thyroid to cortex connection through *tomm70a* which orchestrates a multi-meric protein complex formation that drives apoptosis/mitophagy (Fig. 6). Connection (i) is associated with acetylcholine and neurite outgrowth. This is in accordance with our hypothesis and other studies^13,48–50^ that transient hypothyroidism impacts acetylcholine pathway and the apoptotic pathway. Connection (ii) is associated with neurodegenerative diseases including Alzheimer and Parkinson^51^.

These directed biochemical reactions contrast with the non-directed statistical inferred networks by improving the mechanistic understanding of the integrated response to repetitive ITB. Indeed, the statistical inferred Bayesian and Diablo networks are by nature dedicated to highlight correlations and not causalities. Furthermore, these networks are undirected networks solely based on gene expression.

In contrast to the previous networks, the PD diagram shows a directed network suggesting a putative mechanism based on empirical data. Indeed, the different components of this network are extracted from publications, available on PubMed, that support interactions between the unmeasured molecules from both organs and the candidate genes provided by the statistical analyses.

This study demonstrates the dimensional reduction of the data obtained by the proposed approach: starting from large-scale of measured genes in thyroid and cortex towards a reduced number of candidates potentially involved in the interconnection between cortex and thyroid (Fig. 5). However, these results play only a part in a more complex physiological process including endocrinology, metabolism, etc.

### Final conclusion

Our vigorous work flow is to our knowledge the first that combines advanced statistical network inference approaches with biochemical network validation which establishes a thyroid/cortex integrated molecular mechanism between acetylcholine pathway, neurogenesis, and mitophagy in a repetitive ITB in utero framework. In addition, the intra-organ PD networks show the implication of apoptosis and acetylcholine synthesis in the thyroid and cortex, respectively. This study may provide new insights for improvement in the management of adverse effect of ITB for the protection of the foetuses.

## Materials and methods

### Protocol experiments

Animals were housed at the IRSN animal facilities that have been accredited by the French Ministry of Agriculture to perform experiments on rodents. Animal experiments were conducted in compliance with the French and European regulations for the protection of animals used for scientific purposes (EC Directive 2010/63/EU and French Decree 2013–118). All experimental procedures were approved by the Animal Care Committee of the institute of radioprotection and nuclear safety, and complied with the French regulation for animal experimentation (Ministry of agriculture Act No.87-848, October 19th 1987, modified May 20th 2001; APAFIS#10827-2017073121562679 v2).

The 0.9% NaCl (pH 7.4) and KI (1 mg/kg) solutions were prepared and kindly provided by the Central Pharmacy of Armed forces (Orleans, France).

The experimental procedure was performed as described before^8^. In short, the study included sixteen pregnant Wistar rats (at third day of gestation) that were provided by Charles River at (Charles River laboratories, L’arbresle, France), divided into control group receiving 0.9% NaCl solution, and treated group receiving 1 mg/kg KI for eight days beginning at the ninth gestational day until the 16^th^ gestational day. Both solutions were administered by gavage. The rats were housed individually upon arrival and allowed to recover from transportation until the 9^th^ gestational day. Rats were kept in regular light/dark schedule (12 h/12 h), at 21 ± 2 °C and 50 ± 10% humidity. Food 0.3 mg I/kg of pellet (A04-10 SAFE, Augy, France) and water were freely accessible. After the birth and the weaning, male pups were separated from their mothers; after that, they were divided into control progeny that are not exposed in utero to KI, and treated progeny, exposed in utero to KI; each group included thirteen animals.

Thirty days after the weaning, thyroid and cerebral cortex from the progeny were collected and instantly deep-frozen in liquid nitrogen and then stored at -80 °C until use.

### RNA extraction and quality control

Total RNA was extracted from both tissues, thyroid and cortex respectively, using mirVana™ Isolation Kit (ThermoFisher Scientific, France) according to the manufacturer’s protocol.

RNA quality control was performed using the kit RNA 6000 nano (Agilent Technologies, Waldbronn, Germany) on an Agilent 2100 Bioanalyzer System (Agilent Technologies, Waldbronn, Germany). Quality of RNA was defined by a 260/280 absorbance ratio > 1.8; a 260/230 ratio > 1.5 and a RIN value > 7.5 were desired.

### Transcriptomics

Transcriptomics on thyroid and cortex was routinely performed by CRIBIOM (Marseille, France) using the One-colour Microarray-Based gene expression analysis, according to the low input Quick Amp labelling version 6.9.1 protocol (Agilent Technologies, Waldbronn, Germany). An amount of 100 ng of total RNA was hybridised with Cy-3 and subsequently hybridised on microarrays (SurePrint G3 8 × 60 K, Agilent). The raw fluorescence signals were scanned with DNA Microarray Scanner SureSelect (Agilent Technologies, Waldbronn, Germany) and analysed with the R-package Limma^52^.

### Network inference

The Gene network inference was performed using the R-package Shrinknet v1.0 with parameters suggested by the authors^53^ using expression data obtained from transcriptomics. ShrinkNet calculates the interaction (edges) between genes/nodes^53^ and a set of edges defines the topology of a network which may generate useful hypothesis about the adverse effect of repetitive ITB. Only edges with an FDR lower than 0.05 were kept to construct the network.

### sPLS-DA and DIABLO framework

Besides the previous gene network inference approach, a supervised multivariate analysis was conducted by means of the R package mixOmics (version 6.6.1) in R studio^54^ to specifically target inter-group differences (KI and Controls). Two sparse partial least square discriminant analysis (sPLS-DA) allows the selection of the most predictive or discriminative features in the data to classify the samples accordingly^55^. sPLS-DA helps to prioritise genes of interest for the construction of the PD network.

Firstly, sPLS-DA models were conducted separately on cortex and thyroid transcriptomics’ result. After this intra-organ analysis, an inter-organ transcriptomics integration was conducted via the DIABLO framework^56^. DIABLO constructs components across the cortex and thyroid transcriptomics matrices, maximizing their covariance given a control or KI treatment group as a response variable. These components are defined as linear combinations of transcriptomics variables and a L1 penalization ensures their sparsity. In all the analyses (inter and intra organ), To establish the model parameters including the number of components and the number of features per component the Leave-One-Out Cross validation method was used.

The results were carried out in order to obtain a relevance network (Correlation threshold = 0.82) using the “network” command and permits the selected features representation and highlight positive or negative association in the within/between organ connections.

The cytoscape software^57^ v.3.420 was used to visualize the networks obtained by all the network inference approaches (Shrinknet, sPLS-DA and DIABLO).

### PD Network construction

The manual construction of a biochemical reaction network commenced from the collection of common genes, highlighted simultaneously by the methods described in the network inference section above (Shrinknet, sPLS-DA and DIABLO), assuming that it constitutes the most robust significant gene candidates. The construction of the PD networks was performed as described before^58^; to enrich the inferred networks with relevant information from scientific articles, articles were retrieved from the PubMed database using the molecule(s) of interest as key word(s), *e.g.* “gene A” was used as a query or the combination either “thyroid” or “cortex”, respectively, and “gene A” was used as query. “Gene B” was used as separate query either in combination with key words or not. No data-mining tools were used. The CellDesigner software (version 4.4)^59^ was used to represent molecular biological mechanisms, derived from the literature, resulting in a structured network representation (PD diagram) compliant with Systems Biology Markup Language (SBML) level 2 that is suitable for further computational analysis^60^. Each reaction has been annotated in the “reaction note” at least once with a corresponding scientific article. The CellDesigner’s graphical notation^61^ can be converted into the Systems Biology Graphical Notation (SBGN) standard^62^ by using the CellDesigner’s internal convertor. For each PD diagram, the CellDesigner software creates an xml-file that can be retrieved from the supplementary material section. In addition, a svg-file format from a PD network can be downloaded instead which can be opened by any internet browser and the diagram can viewed with different zoom levels. These files are available from the supplementary material section as well.

### Colouring molecules in the PD network according to gene expression

The genes that are differentially regulated (transcriptomic analysis) and are present in the PD network can be coloured according to their log2 FC values by using the Cytoscape plugin BiNoM^63^ with Cytoscape v2.8. With the BiNoM plugin the CellDesigner file can be imported into the Cytoscape environment and colours can be assigned to genes and proteins according to their fold change.

## Supplementary information

Supplementary file1 (SVG 152 kb)

Supplementary file2 (SVG 470 kb)

Supplementary file3 (SVG 227 kb)

Supplementary file4 (XLSX 22 kb)

Supplementary file5 (XLSX 19 kb)

Supplementary file6 (XLSX 32 kb)

## Data Availability

The datasets generated during and/or analysed during the current study are available in the Gene Expression Omnibus repository, accession number GSE148281, https://www.ncbi.nlm.nih.gov/geo/query/acc.cgi?acc=GSE148281.
